# APOE-knockout in rabbits causes loss of cells in nucleus pulposus and enhances the levels of inflammatory catabolic cytokines damaging the intervertebral disc matrix

**DOI:** 10.1371/journal.pone.0225527

**Published:** 2019-11-21

**Authors:** Anja Beierfuß, Monika Hunjadi, Andreas Ritsch, Christian Kremser, Claudius Thomé, Demissew Shenegelegn Mern

**Affiliations:** 1 Laboratory Animal Facility, Medical University of Innsbruck, Innsbruck, Austria; 2 Department of Internal Medicine I, Medical University of Innsbruck, Innsbruck, Austria; 3 Department of Radiology, Medical University of Innsbruck, Innsbruck, Austria; 4 Department of Neurosurgery, Medical University of Innsbruck, Innsbruck, Austria; Max Delbruck Centrum fur Molekulare Medizin Berlin Buch, GERMANY

## Abstract

Rabbits with naturally high levels of cholesterol ester transfer protein (CETP), unlike rodents, have become an interesting animal model for the study of lipid metabolism and atherosclerosis, as they have similarities to humans in lipid metabolism, cardiovascular physiology and susceptibility to develop atherosclerosis. Rodents, such as mice, are not prone to atherosclerosis as they lack the mass and activity of CETP, as a key player in lipoprotein metabolism. Recently, APOE-knockout in rabbits has been shown to promote atherosclerosis and associated premature IVD degeneration that mimic the symptoms of atherosclerosis and structural changes of IVDs in humans. Here we examined whether APOE-knockout promoted IVD degeneration in rabbits is associated with imbalanced inflammatory catabolic activities, as the underlying problem of biological deterioration that mimic the symptoms of advanced IVD degeneration in humans. We analysed in lumbar nucleus pulposus (NP) of APOE-knockout rabbits the cell viabilities and the intracellular levels of inflammatory, catabolic, anti-catabolic and anabolic proteins derogating IVD matrix. Grades of IVD degeneration were evaluated by magnetic resonance imaging. NP cells were isolated from homozygous APOE-knockout and wild-type New Zealand White rabbits of similar age. Three-dimensional cell culture with low-glucose was completed in alginate hydrogel. Cell proliferation and intracellular levels of target proteins were examined by MTT and ELISA assays. Alike human NP cells of different disc degeneration grades, NP cells of APOE-knockout and wild-type rabbits showed significantly different in vivo cell population densities (p<0.0001) and similar in vitro proliferation rates. Furthermore, they showed differences in overexpression of selective inflammatory and catabolic proteins (p<0.0001) similar to those found in human NP cells of different disc degeneration grades, such as IL-1β, TNF-α, ADAMTS-4, ADAMTS-5 and MMP-3. This study showed that premature IVD degeneration in APOE-knockout rabbits was promoted by the accumulation of selective inflammatory catabolic factors that enhanced imbalances between catabolic and anabolic factors mimicking the symptoms of advanced IVD degeneration in humans. Thus, APOE-knockout rabbits could be used as a promising model for therapeutic approaches of degenerative disc disorders.

## Introduction

Intervertebral disc degeneration is one of the main causes of low back pain. It is characterized by structural deterioration and unfavourable changes in molecular phenotype of IVD cells that enhance expression levels of inflammatory cytokines, such as interleukin beta (IL-1β) and tumour necrosis factor alpha (TNF-α). Inflammatory cytokines have been described to induce inflammatory catabolic processes in IVDs and promote accelerated degradation of the extracellular matrix [1–4]. Successive imbalanced inflammatory catabolic processes in IVDs evidently cause progressive chronic back pain, which is one of the most prevalent musculoskeletal disorders affecting a vast majority of adults over 30 years old. Progressive chronic back pain that promotes disabilities and social isolation causes huge socio-economic costs in terms of medication, disability benefits and lost productivity [5–7]. Various factors, such as abnormal biomechanical loading, aging, genetic predisposition, smoking, infection and declined nutrient transport into IVDs, have been described to induce enhanced expression of inflammatory and catabolic factors [8–14]. Although the relative importance and the interrelationships among each of these factors are not yet clearly known, each of these factors contributes to the progression of IVD degeneration [8–15].

Nucleus pulposus is located in the centre of the avascular IVD around 8 mm apart from the nearest blood supply. Cells in NP tissue receive nutrition from the surrounding blood vessels of the vertebral body by diffusion, which occurs due to concentration gradients set up by cellular metabolism [16]. Impairment of nutrient transport into IVDs can lead to declined concentration of glucose, pH and oxygen (pO_2_) that adversely affects the activities as well as the survival of IVD cells, especially NP cells in the middle of the IVDs. Accordingly, nutrient impairment is considered as one of the major factors of IVD degeneration [16–19]. Atherosclerosis that can obstruct the abdominal aorta and its branching lumbar arteries supplying the vertebrae with nutrients could weaken the nutrient transport into IVDs. Mature atherosclerotic plaques obstructing the abdominal aorta and lumbar arteries have been found in patients with low back pain and degenerative disc disorders [20–25]. Both atherosclerosis and IVD degeneration show interrelated emerging and advancing processes: they begin at an early adult age and their rapid progression follow between 44 and 64 years of age [26–27, 13].

Deficiency of APOE promotes type III hyperlipoproteinemia (HLP) and supports the development of premature atherosclerotic plaques [28–31]. APOE-knockout in rabbits has been shown to abnormally elevate the levels of plasma cholesterol, triglycerides and remnant lipoproteins and induce excessive aortic atherosclerosis that mimic the symptoms of cardiovascular disease in humans [32–33]. In addition, we recently have shown in atherosclerotic APOE-knockout rabbits the impairment of nutrient supply into IVDs, which led to declined glucose concentration, loss of cell viability and premature degeneration [34]. NP cells play a decisive role in IVD homeostasis by orchestrating the expressions and activities of inflammatory, catabolic, anti-catabolic and anabolic factors that derogate the extracellular matrix of IVDs. The degree of imbalances between those factors has been shown to be highly correlated with the grades of IVD degeneration in humans [35–36]. Human NP cells have shown degeneration grade dependent accumulations of selective inflammatory and catabolic proteins that are accompanied by low expression levels of anabolic factors [35–36]. As the effect of APOE-knockout on the phenotypic changes derogating the extracellular matrix of IVDs has not yet been investigated, we examined the protein expression patterns of therapeutic relevant inflammatory, catabolic, anti-catabolic and anabolic factors in NP cells of APOE-knockout rabbits. We assessed whether the accelerated IVD degeneration in APOE-knockout rabbits is associated with the accumulations of selective inflammatory and catabolic cytokines that may mimic the symptoms of advanced IVD degeneration in humans. The expression patterns of 28 target proteins were determined in NP cells of six APOE-knockout and six wild-type New Zealand White rabbits of age about two-years. We discussed the unfavourable phenotypic changes found in NP cells of APOE-knockout rabbits in relation to that found in NP cells of degenerative human IVDs. APOE-knockout rabbits would be used as a favourable *in vivo* model for rational therapeutic approaches of degenerative disc disorders.

## Materials and methods

### Ethics statement and animals

The Laboratory Animal Facility at the Medical University of Innsbruck and all experimental procedures of the study were complied with the Austrian Animal Experimental Act (BGBI. I Nr. 114/2012). The approvals of the Laboratory Animal Facility (BMWFW-66.011/0017-II/3b/2014) and the experimental procedures were obtained from the National Committee for Animal Care of the Austrian Federal Ministry of Education, Science and Research. APOE-knockout rabbits were created and homozygous APOE-knockout mutants were made available [32] and bred by the Laboratory Animal Facility at the Medical University of Innsbruck. Six male APOE-knockout NZW rabbits (mean age 2.0 ± 0.2 years; mean weight 4.4 ± 0.3 kg) and six male wild-type NZW rabbits (mean age 2.0 ± 0.3 years; mean weight 4.0 ± 0.2 kg) were used for the study. Each rabbit was single housed in a flat deck cage (5400 cm^2^, Scanbur, Denmark) with an elevated platform. Housing conditions were maintained at 18 °C, 50% relative humidity and 12/12 hours light/dark cycle. Specific Pathogen Free (SPF) quality of the animals was monitored and confirmed according to FELASA recommendations [37]. Commercial standard diet (K-H/V223X, Ssniff^®^, Germany) was fed ad libitum and fresh tap water was constantly available. Regular health monitoring and an additional health check prior to anaesthesia was performed by the responsible veterinarian.

### Rabbit lumbar spine MRI

Magnetic resonance imaging (MRI) was established to assess the degeneration grades of the rabbit lumbar IVDs [34]. Briefly, ahead of MRI a rabbit was sedated by intramuscular injection of ketamine (35 mg/kg body weight, Ketasol^®^, aniMEDICA GmbH, Germany) and xylazine (5 mg/kg body weight, Xylasol^®^, aniMEDICA GmbH, Germany). Additionally, a 22-gauge intravenous catheter was placed into a marginal ear vein and a constant infusion of ketamine (130 mg/kg) and xylazine (8 mg/kg) in 0.9% saline of 100 ml mini-bag (B. Braun GmbH, Germany) was administered at a rate of 0.15 ml/min, which provided sufficient anaesthesia. There was a constant video monitoring of the anesthetized animal during MRI analysis. MRI of the lumbar spine was performed using a 3T whole-body MRI scanner (Magnetom Verio, Siemens Healthcare). The rabbit was positioned prone on a 24 channel spine array coil and covered with a flexible 8-channel body array coil. Images were acquired in sagittal orientation. T2-weighted (T2w) images were obtained with a fast-spin-echo (FSE) sequence (T2-FSE) (TR/TE = 3000ms/53ms, echo train length: 12, acquisition matrix: 320x236, rectangular FOV: 140mm x 103mm, number of slices: 13, slice thickness: 2mm, interslice gap: 0.2mm, number of averages: 4, voxel size: 0.44mm x 0.44mm x 2mm). The Pfirrmann MRI scoring system was used for evaluation of lumbar disc degeneration on T2w images [38]. The Pfirrmann degenerative grade criterial and degree of degeneration in brief. Grade I: The structure of the disc is homogeneous, with a bright hyperintense white signal intensity and a normal disc height. Grade II: The structure of the disc is inhomogeneous, with a hyperintense white signal. The distinction between nucleus and anulus is clear, and the disc height is normal, with or without horizontal gray bands. Grade III: The structure of the disc is inhomogeneous, with an intermediate gray signal intensity. The distinction between nucleus and anulus is unclear, and the disc height is normal or slightly decreased. Grade IV: The structure of the disc is inhomogeneous, with an hypointense dark gray signal intensity. The distinction between nucleus and anulus is lost, and the disc height is normal or moderately decreased. Grade V: The structure of the disc is inhomogeneous, with a hypointense black signal intensity. The distinction between nucleus and anulus is lost, and the disc space is collapsed.

### Recruitment of NP tissue from rabbit intervertebral discs

Rabbits were deeply sedated by intramuscular injection of ketamine (35 mg/kg body weight, Ketasol^®^, aniMEDICA GmbH, Germany) and xylazine (5 mg/kg body weight, Xylasol^®^, aniMEDICA GmbH, Germany) and euthanized by intracardiac administration of concentrated potassium chloride. Lumbar discs were immediately harvested. NP tissue was carefully separated from anulus fibrosus (AF) tissue on the basis of their macroscopic morphology (identification of the innermost lamellar ring of the AF). To avoid contamination of the NP samples great care was taken to exclude surrounding tissues and blood. The NP tissue (L1/L2-L6/S1) of each rabbit was separately pooled for isolation of NP cells.

### Isolation of NP cells

Samples of the pooled NP tissues were separately and finely minced into small fragments of approximately 2 mm^3^. From each rabbit a 200 mg portion of finely minced NP tissues were separately digested for direct determination of viable cell concentration per mg of NP tissue. The rest of finely minced NP tissues from each rabbit was separately digested for culturing of NP cells. Samples were directly digested with 0.02% w/v pronase (Sigma-Aldrich) in 5 ml DME/F-12 (Dulbecco’s Modified Eagle’s Medium/Ham’s Nutrient Mixture F12, 1:1 mixture) containing 1% penicillin/streptomycin, 50 mg/mL L-ascorbic acid, 1% glucose and 10% FCS (fetal calf serum) (Sigma-Aldrich) (1 h, 37 °C, 5% CO_2_). After filtration of the samples through sterile 75 gm nylon mesh filters (Sigma-Aldrich) and centrifugation of the supernatants (1000 x g, 2 min), pellets were resuspended in 5 ml DME/F-12 and digested with 0.02% w/v collagenase II (Sigma-Aldrich) (3 h, 37 °C, 5% CO_2_). Samples were filtered through sterile 75 gm nylon mesh filters, supernatants were centrifuged (1000 x g, 2 min). The pellets of the 200 mg portions were separately resuspended in 1 ml DME/F-12 for direct application of MTT assay. Pellets of the rest samples were separately resuspended in 3 ml DME/F-12 containing 1% penicillin/streptomycin, 1% glucose and 10% FCS and sequentially expanded in Greiner CELLSTAR dishes (35 mm x 10 mm) and 25 cm^2^ flasks (Sigma-Aldrich) for four weeks (37 °C, 5% CO_2_) by changing the culture medium every two days. NP cells were cryopreserved at -196 °C in culture medium containing 30% FCS and 15% dimethyl sulfoxide (DMSO) (Sigma-Aldrich).

### Three dimensional culture of NP cells

For three dimensional (3D) cell culture the Alginate 3D Cell Culture Kit (AMSBIO) was used according to the instruction of the manufacturer. Briefly, 50 ml calcium chloride solution was dispensed in a sterile 100-ml beaker containing a sterile stir bar; and NP cell pellet containing 1 x 10^6^ cells was prepared in 15-ml Eppendorf tube after harvesting the cells using Trypsin-EDTA (Sigma-Aldrich). Then 5 ml sodium alginate solution was dispensed into the 15-ml tube and mixed with a pipette to prepare homogeneous cell suspension. The cell suspension was aspirated into a 5-ml syringe that was attached with a plastic flexible needle. After removing the plastic flexible needle, a 22G hypodermic needle was attached to the syringe to drop the cell suspension into the beaker containing the calcium chloride solution. The cell suspension was added into the stirring calcium chloride solution at about two drops per second by holding the syringe in an upright position over the beaker and by positioning the tip of the needle about 5 cm above the liquid surface of calcium chloride. Following dropping, the beads in calcium chloride solution were stirred for further 10 min until the alginate beads have coagulated and appeared completely white. The calcium chloride solution was removed using a manual pipette without aspirating the beads and 100 ml saline solution was added to the beads. The saline solution was removed after 15 min using a manual pipette and the beads were mixed for 10 min with 100 ml culture medium. After removing the medium, 10 alginate beads were scooped using a sterile spatula and placed into a separate well of the 24-well plate containing 2 ml culture medium. NP cells were cultured in 24-well plates for 4 weeks (37 °C, 5% CO_2_) by changing the culture medium every two days. For each sample three independent 3D cultures were performed.

### Recovery of NP cells from alginate beads

The 3D culture medium was removed from the entire well using a manual pipette. Alginate beads were dissolved by adding 1 ml of sodium citrate solution to each well and mixing at room temperature for 10 min. The alginate solution of the 24-well plates was transferred to 50-ml tube and centrifuged at 1000 ×g for 2 min. Cells were then harvested as a precipitated pellet and prepared for analysis of cell proliferation or protein concentration.

### Determining the number of viable NP cells

To determine the number of viable cells in NP tissues and 3D alginate cultures, the cell proliferation assay kit MTT (3-(4,5-dimethylthiazol-2-yl)-2,5-diphenyltetrazolium bromide) was used according to the manufacturer’s protocol (Invitrogen Molecular Probes). Briefly, duplicates of 100 μl samples and duplicates of blanks (100 μl medium alone) were plated into a flat-bottomed 96 well plate and incubated to recover the cells from handling (24 h, 37 °C, 5% CO_2_). After adding 10 μl MTT Reagent to each well and incubation for 3 h (37 °C, 5% CO_2_), 100 μl of the SDS-HCl solution was added and further incubated for 4 h (37 °C, 5% CO_2_). A microtiter plate reader AF 2200 (Eppendorf) was used to measure the absorbance in each well at 570 nm. The average value of the blank duplicate readings was subtracted from the average values of the sample duplicate readings and the number of viable cells was calculated from the standard curve. For each sample at least three independent assays were performed with two replicates.

### Total protein extraction and quantification

Whole protein extraction from 3D cultured NP cells was performed using radio-immunoprecipitation assay (RIPA) buffer (Sigma-Aldrich). NP cell pellets were washed twice in cold PBS (2500 x g, 5 min) and resuspended with 300 ml cold RIPA buffer containing protease inhibitor and phosphatase inhibitor cocktails (Sigma-Aldrich). After sonication (30 sec, 50% pulse) the mixture was shacked gently on ice (15 min) and centrifuged (14000 x g, 15 min and 4 °C). Supernatants were transferred to new tubes for protein quantification. Total protein concentration in samples was determined by using Pierce Micro BCA Protein Assay Kit according to the instruction manual (Thermo Scientific).

### Enzyme-linked immunosorbent assay

To determine the presence and concentration of target proteins in rabbit NP cells Enzyme-linked immunosorbent assays (ELISAs) were applied on 100 μg of total protein extracts. For measuring the concentration of target proteins ELISA kits were purchased from LifeSpan BioSciences (USA), Neo Scientific (USA), ARP American Research Products (USA), R & D Systems (United Kingdom) and Uscn Life Science (USA). Assays were performed according to the instruction manuals of the kits. The investigated proteins include: inflammatory cytokines such as IL-1β (interleukin-1β), IL-1 R1 (interleukin-1 receptor), TNF- α (tumour necrosis factor-alpha) and TNF-R1(tumour necrosis factor receptor 1); catabolic factors such as ADAMTS-4, ADAMTS-5 (a disintegrin and metalloproteinase with thrombospondin motifs), MMP-1, MMP-2, MMP-3, MMP-7, MMP-8, MMP-9, MMP-10 and MMP-13 (matrix metalloproteinase); anti-catabolic factors such as TIMP-1, TIMP-2, TIMP-3 and TIMP-4 (tissue inhibitors of metalloproteinases); anabolic factors such as BMP-2, BMP-4, BMP-6, BMP-7 (bone morphogenetic proteins), IGF-1 (insulin-like growth factor 1), TGF-β1 and TGF-β3 (transforming growth factor beta); matrix proteins such as aggrecan, collagen II and I; in addition APOE in wild-type. At least three independent assays were done for each sample with two replicates.

### Quantitative reverse transcription PCR

The quantitative reverse transcription PCR (RT-qPCR) was applied to examine the mRNA expression of APOE in NP cells of wild-type rabbits. From 3D cultured NP cells total RNA was isolated by using the RNeasy Plus Mini Kit (Qiagen). DNA contamination was removed by DNase 1 (Sigma-Aldrich). The concentration of total RNA was quantified at 260 nm using Biospectrometer (Eppendorf) and equal amounts of RNA were used for reverse transcription (RT). The cDNAs were synthesized using TaqMan Reverse Transcription Reagents (Applied Biosystems) and the mRNA levels of APOE and β-Actine (internal standard) were determined by qPCR using TaqMan gene expression assay (Life Technologies) and LightCycler 480 (Roche Applied Science). The TaqMan Gene Expression Master Mix (1× master mix) supplemented with 200 nM sense, 200 nM antisense primers of APOE, 250 nM APOE-probe and 2 μl of the template DNA was used for PCR reactions in 20 μl of final volume. The PCR program contained an initial denaturation step at 95 °C for 15 min, 40 cycles of denaturation at 95 °C for 15 s, an extension at 60 °C for 1 min, a melt curve stage (65 °C to 95 °C, increment 0.5 °C) and a final extension at 72 °C for 10 min. Standard, negative control and sample were run in three replicates of a 96 well plate. The mRNA expression levels were numerically presented using the delta CT (ΔCT) method. For each sample three independent assays were performed with two replicates. The (5’->3’) sequences of primers and probes are:

APOE-sense: AGGAGCTGACCATGCTGATG
APOE-antisense: CTGTTGCACACGTCCTCCAT
APOE-probe: 6FAB-CCATGCTGATGGAGGAGACC-BHQ1
Beta-actin-sense: CAGAAGGACAGCTACGTGGG
Beta-actin-antisense: CATGTCGTCCCAGTTGGTCA
Beta-actin-probe: 6FAB-GACCCTGAAGTACCCCATCG-BHQ1

### Statistical data analysis

MRI Scans were analysed independently by two observers. Agreement percentage of two observers (within observers: intraobserver reliability, and between observers: interobserver reliability) was applied for reliable MRI evaluations of lumbar IVD degeneration according to Landis and Koch based interpretations of κ statistics [38–39]. Statistical data analyses were performed by the use of IBM SPSS Statistics 22 (Armonk New York USA). For each sample at least three independent assays were done. 1-way ANOVA and pairwise comparisons were used to compare the data of APOE-knockout and wild-type rabbits. Significance was set at p < 0.01.

## Results

### Interobserver reliability of intensified IVD degeneration in APOE-knockout rabbits

**Fig 1:**
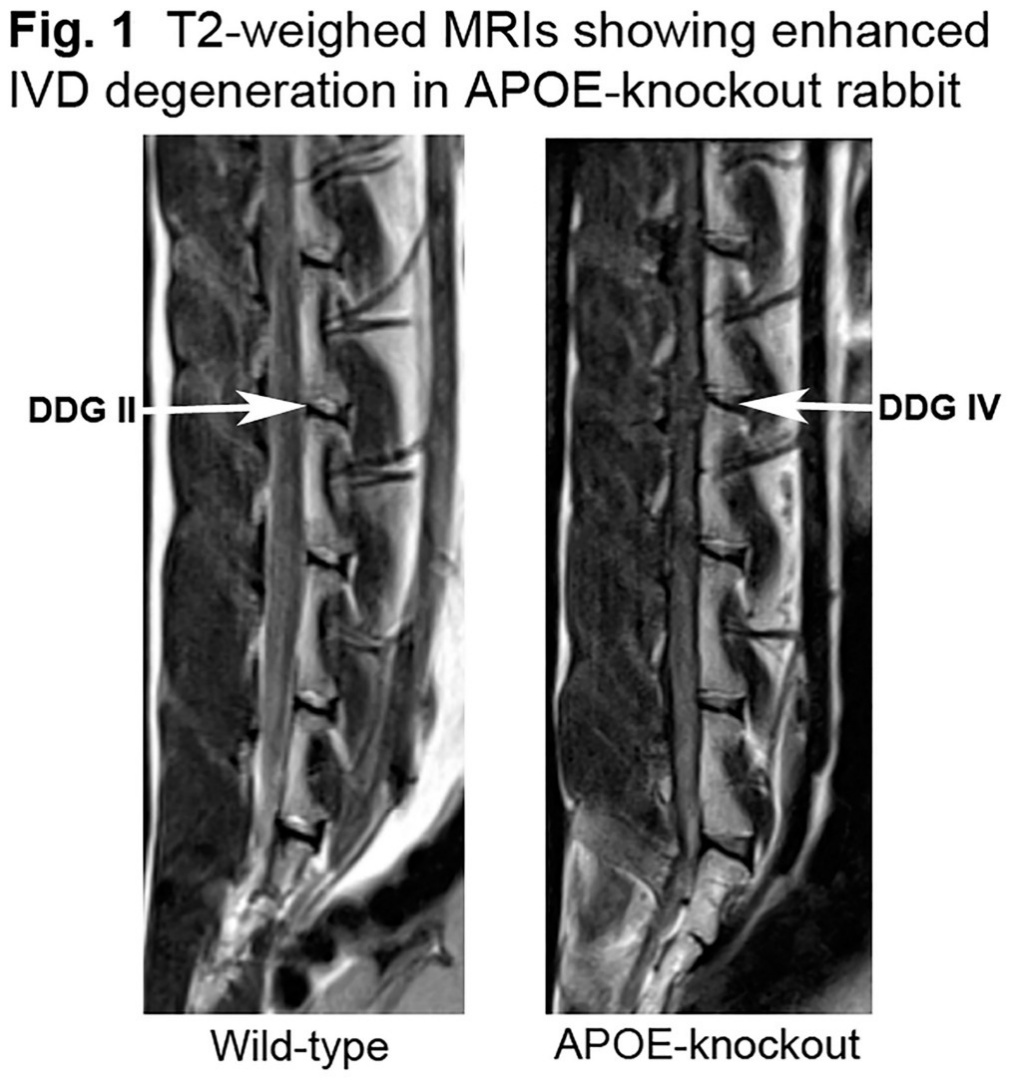
Representative T2 weighted IVD images illustrating lumbar IVDs from two years old wild-type and APOE-knockout rabbits. The MRIs displayed considerably enhanced disc degeneration in the APOE-knockout rabbit with disc degeneration grade IV (DDG IV) as compared to DDG II in wild-type rabbits. Arrows showing IVDs.

Interobserver reliability agreement for rating intervertebral disc degeneration with two observers was done as described before [38–39]. The T2-weighted MRI signal characteristics of the lumbar IVDs showed substantially enhanced IVD degeneration in APOE-knockout rabbits as compared to that in the wild-type rabbits. The calculated frequency of interobserver reliability disagreements were 0.00% with a reliability coefficient κ = 1.00. Higher score of IVD degeneration grade was confirmed in APOE-knockout rabbits. Representative T2 weighted IVD images of wild-type and APOE-knockout rabbits are shown in Fig 1.

### *In vivo* loss of NP cells in APOE-knockout rabbits

**Fig 2:**
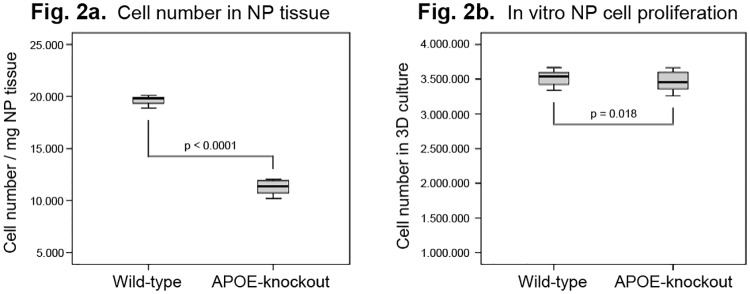
Cell number in NP tissue and *in vitro* NP cell proliferation of APOE-knockout rabbits. For determination of *in vivo* NP cell population density, cells were isolated from NP tissues immediately after euthanization of the rabbits for direct determination of cell numbers per mg of NP tissue. For determination of *in vitro* cell proliferation rate 1 x 10^6^ NP cells were cultured four weeks in alginate gel and viable cells were quantified using MTT assays.

To determine the *in vivo* population density of NP cells, cells were isolated from NP tissues immediately after euthanazia of the rabbits and MTT assays were directly applied to quantify the numbers of viable NP cells. Diminished numbers of NP cells per milligram of wet tissue [cell number/milligram tissue] were ascertained in NP tissues of APOE-knockout rabbits. The recorded mean numbers of NP cells were 11400 ± 681 for APOE-knockout rabbits and 19619 ± 406 for wild-type rabbits (p<0.0001). As compared to the mean values of the wild-types the amount of NP cells in APOE-knockout rabbits was reduced by about 41.7% (Fig 2a). However, the three dimensional culture of 1 x 10^6^ NP cells in alginate gel for four weeks showed similar proliferation rates for all samples of APOE-knockout and wild-type rabbits. The comparable mean NP cell numbers were 3464578 ± 130332 and 3513561 ± 96851 for APOE-knockout and wild-type rabbits respectively (p = 0.018) (Fig 2b).

### Levels of inflammatory and catabolic cytokines in NP cells of APOE-knockout rabbits

Since inflammatory cytokines can enhance the expression of catabolic factors and promote an inflammatory catabolic metabolism of the IVD matrix, we determined the levels of inflammatory and catabolic proteins in NP cells of APOE-knockout and wild-type rabbits.

**Fig 3:**
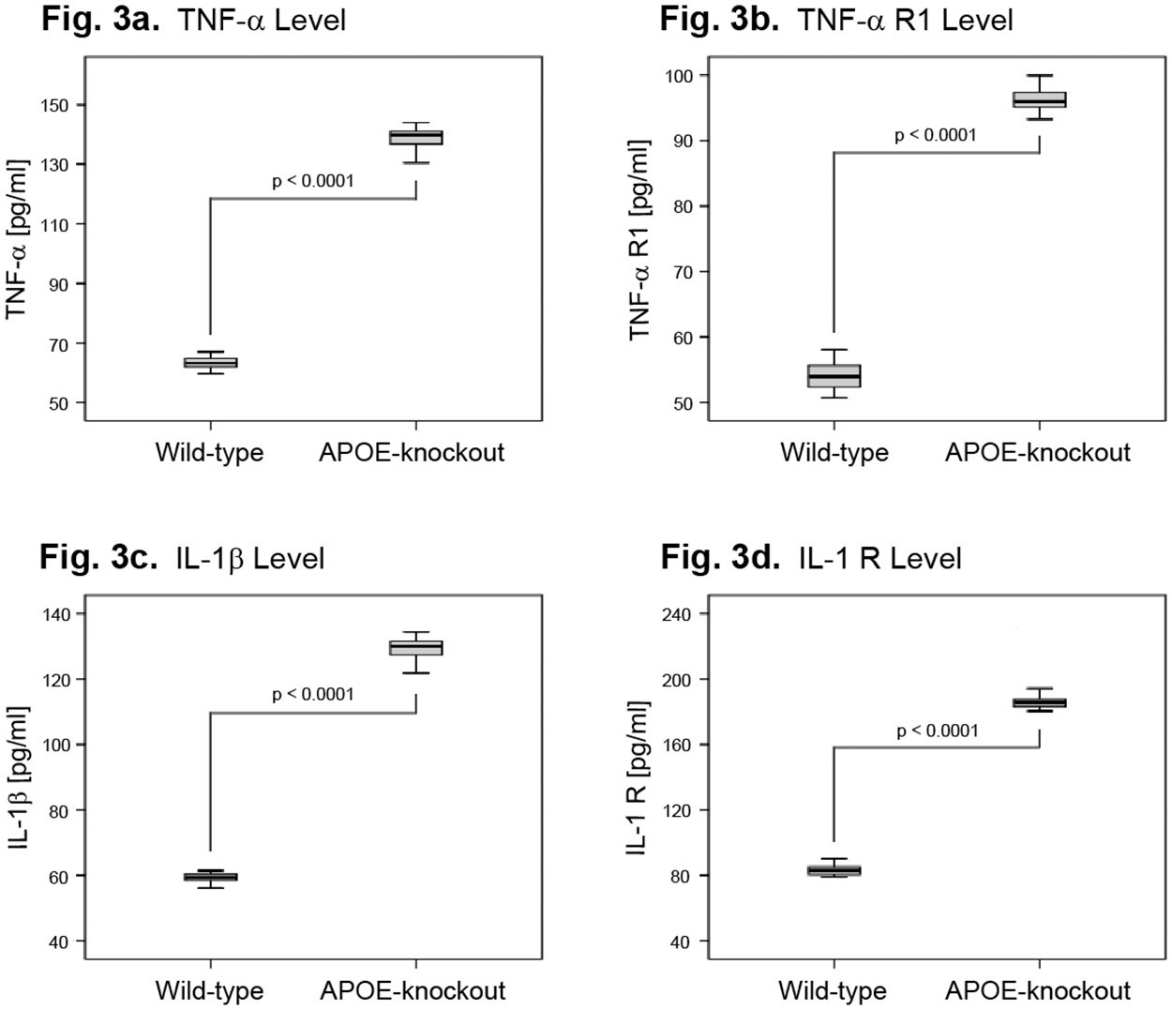
Enhanced levels of inflammatory cytokines in NP cells of APOE-knockout rabbits. From each APOE-knockout and wild-type rabbit 1 x 10^6^ NP cells were cultured in alginate gel for four weeks and the concentrations of inflammatory cytokines in 100 μg total protein extracts were determined (ELISA) and statistically analysed. Box plots with whiskers min. to max. show TNF-α concentration levels (Fig 3a), TNF-α R1 levels (Fig 3b), IL-1β levels (Fig 3c) and IL-1 R levels (Fig 3d).

Enhanced expression levels of the inflammatory cytokines TNF-α and IL-1β as well as their receptors TNF-α R1 and IL-1 R were detected in NP cells of APOE-knockout rabbits. The mean expression levels of TNF-α and TNF-α R1 in NP cells of APOE-knockout rabbits amounted 138.8 ± 3.516 pg/ml and 96.46 ± 2.253 pg/ml respectively; and the respective mean levels for the wild-type rabbits were 63.35 ± 2.016 pg/ml and 54.13 ± 2.127 pg/ml (P<0.0001) (Fig 3a and 3b). The mean expression levels of IL-1β and IL-1 R amounted 129.2 ± 3.288 pg/ml and 185.7 ± 3.495 pg/ml in NP cells of APOE-knockout rabbits; and 59.36 ± 1.407 pg/ml and 83.18 ± 3.192 pg/ml in wild-type respectively (P<0.0001) (Fig 3c and 3d).

**Fig 4:**
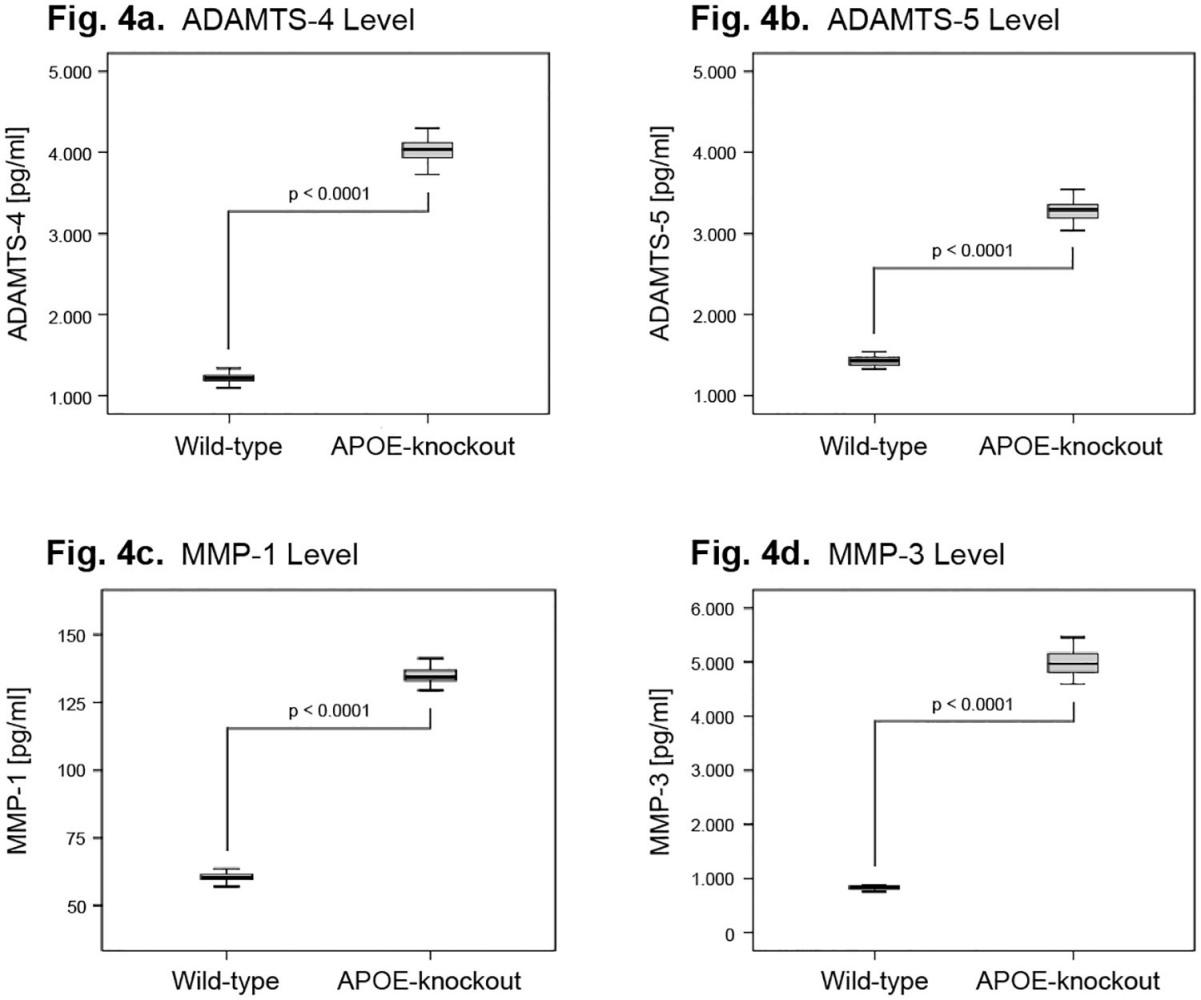
Increased levels of catabolic factors ADAMTS-4/-5 and MMP-1/-3 in NP cells of APOE-knockout rabbits. 1 x 10^6^ NP cells from each APOE-knockout and wild-type rabbit were cultured in alginate gel for four weeks and the concentrations of the catabolic factors were determined (ELISA) in 100 μg total protein extracts and statistically analysed. Box plots with whiskers min. to max. show ADAMTS-4 concentration levels (Fig 4a), ADAMTS-5 levels (Fig 4b), MMP-1 levels (Fig 4c) and MMP-3 levels (Fig 4d).

The catabolic factor ADAMTS-4 and ADAMTS-5 showed high mean expression levels in NP cells of both APOE-knockout and wild-type rabbits. However, the mean levels in NP cells of APOE-knockout rabbits were considerably higher. The mean levels of ADAMTS-4 and ADAMTS-5 were 4017 ± 149.7 pg/ml and 3278 ± 136 pg/ml in APOE-knockout; and 1222 ± 58.28 pg/ml and 1433 ± 60.29 pg/ml wild-type respectively (P<0.0001) (Fig 4a and 4b).

MMPs showed differentiated patterns of expressions in NP cells of APOE-knockout rabbits as compared to that in wild-type. MMP-1 and MMP-3 showed enhanced mean expression levels in NP cells of APOE-knockout rabbits, whereas the mean expression levels of MMP-2, MMP-7, MMP-8, MMP-10 and MMP-13 remained unaffected. The recorded mean expression levels for MMP-1 and MMP-3 were 135.1 ± 2.93 pg/ml and 4967 ± 220.2 pg/ml in APOE-knockout; and 60.32 ± 1.1684 pg/ml and 827.4 ± 34.07 pg/ml in wild-type respectively (P<0.0001) (Fig 4c and 4d). The respective unaffected mean expression levels in NP cells of APOE-knockout and wild-type rabbits were 57.71 ± 2.505 pg/ml and 56.91 ± 1.486 pg/ml (p = 0.168) for MM-2; 190.3 ± 2.376 pg/ml and189.8 ± 2.560 pg/ml (p = 0.515) for MMP-7; 40.11 ± 0.8124 pg/ml and 39.57 ± 0.7512 pg/ml (p = 0.024) for MMP-8; 43.08 ± 0.4889 pg/ml and 42.76 ± 0.9474 pg/ml (p = 0.143) for MMP-10; and 275.2 ± 3.279 pg/ml and 273.3 ± 3.342 pg/ml (p = 0.039) for MMP-13. The concentration level of MMP-9 was not traceable although the kit detection limit was below 100 pg/ml.

### Levels anti-catabolic and anabolic factors in NP cells of APOE-knockout rabbits

**Fig 5:**
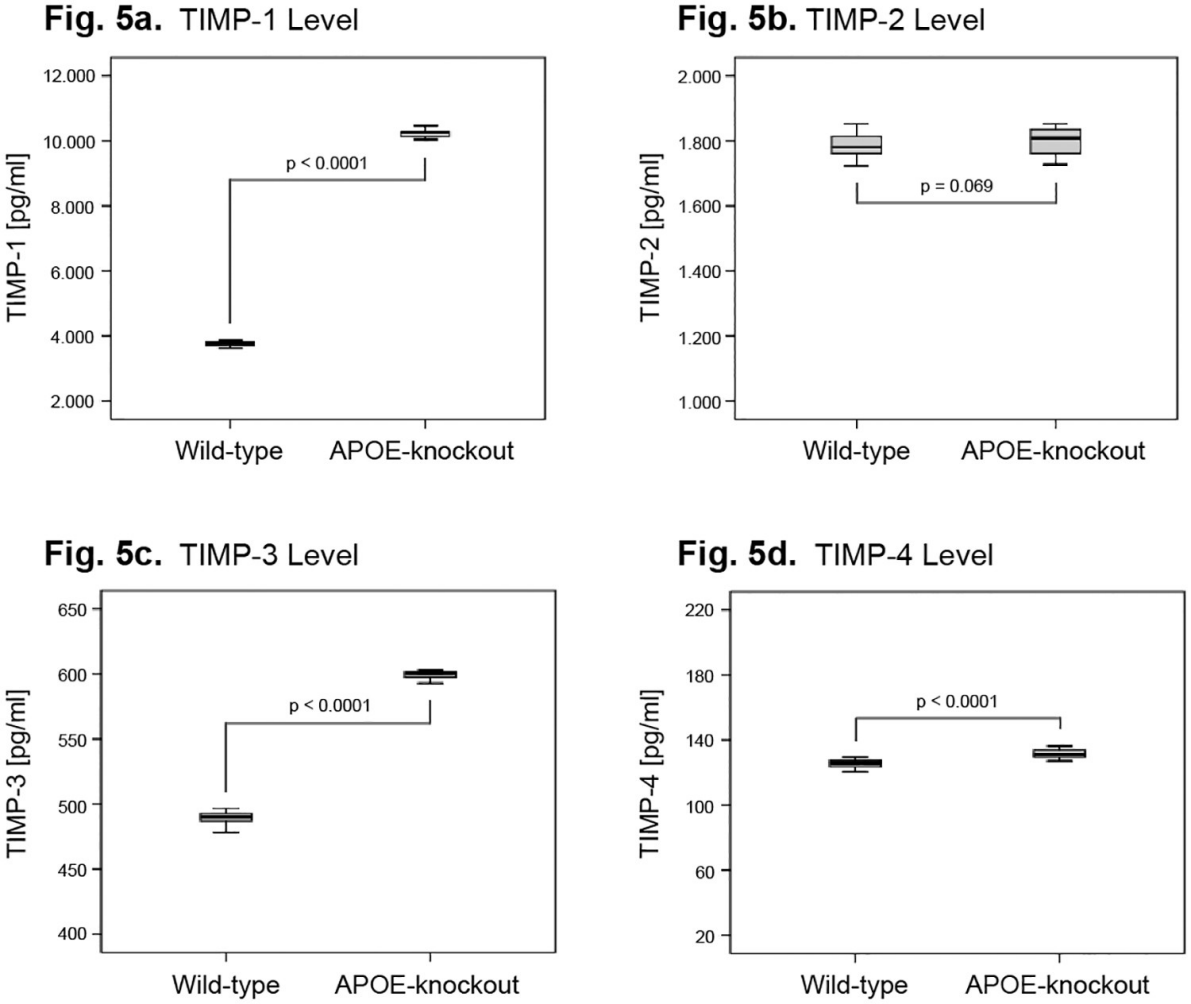
Anti-catabolic TIMPs expression levels in NP cells of APOE-knockout rabbits. Expression levels of the anti-catabolic factors TIMPs were determined (ELISA) in 100 μg total protein extracts isolated from NP cells cultured in alginate gel for four weeks and data were statistically analysed. Box plots with whiskers min. to max. show TIMP-1 levels (Fig 5a), TIMP-2 levels (Fig 5b), TIMP-3 levels (Fig 5c) and TIMP-4 levels (Fig 5d).

Since decreased expression levels of the anti-catabolic factors (TIMPs) can lead to enhanced NP matrix proteolysis, we determined the levels of TIMP-1, TIMP-2, TIMP-3 and TIMP-4 in NP cells of APOE-knockout rabbits. A greatly enhanced level of TIMP-1 was determined in NP cells of APOE-knockout rabbits as compared to that in wild-types. The mean expression levels were 10226 ± 100 pg/ml and 3770 ± 70.39 pg/ml respectively (P<0.0001) (Fig 5a). A moderately enhanced level of TIMP-3 was recorded with mean expression value of 599 ± 2.595 pg/ml in APOE-knockout and 498 ± 5.089 pg/ml in wild-type (P<0.0001) (Fig 5c). TIMP-2 showed equivalent expression levels in both NP cells of APOE-knockout and wild-type rabbits with the mean expression levels of 1799 ± 41.9 pg/ml and 1787 ± 37.91 pg/ml respectively (P = 0.069) (Fig 5b). The lowest and equivalent expression levels were recorded for TIMP-4 with mean levels of 131.5 ± 2.61 pg/ml in APOE-knockout and 125.3 ± 2.606 pg/ml in wild-type (P<0.0001) (Fig 5d).

The expression of several anabolic factors were examined because growth factors could have crucial roles in the development, growth and maintenance of intervertebral discs. From all tested anabolic factors only TGF-β1, IGF-1 and BMP-2 could be detected exclusively in NP cells of APOE-knockout rabbits with mean levels of 51.06 ± 1.518 pg/ml, 63.01 ± 1.615 and 43.34 ± 1.121 pg/ml respectively. Although the minimum detectable level for all tested anabolic factors were very low, the concentration levels of BMP-4, BMP-6 and BMP-7, FGF-2 and TGF-β3 were not traceable in NP cells of both APOE-knockout and wild-type rabbits (Table 1). NP cells of wild-type rabbits did not show any APOE expression as NP cells of APOE-knockout rabbits. This indicates that APOE deficiency in IVDs is apparently not the cause of accelerated IVD degeneration in APOE-knockout rabbits.

**Table 1:** Lack of anabolic factors in NP cells of APOE-knockout rabbits. NP cells were isolated from wild-type and APOE-knockout rabbits. 1 x 10^6^ cells from each sample were grown for four weeks in alginate gel. The protein concentration (ELISA) data in 100 μg total protein extracts were statistically analyzed.

Anabolic proteins	Minimum detectable level [pg/ml]	Level in wild-type		Level in APOE-knockout	
		pg/ml	SD	pg/ml	SD
BMP-2	4.5	43.34	1.231	-	-
BMP-4	0.5	-	-	-	-
BMP-6	2.2	-	-	-	-
BMP-7	0.8	-	-	-	-
FGF-2	1.0	-	-	-	-
IGF-1	7.0	63.01	1.615	-	-
TGF-β1	1.8	51.06	1.518	-	-
TGF-β3	2.4	-	-	-	-

### Deteriorated levels of matrix proteins in in NP cells of APOE-knockout rabbits

**Fig 6:**
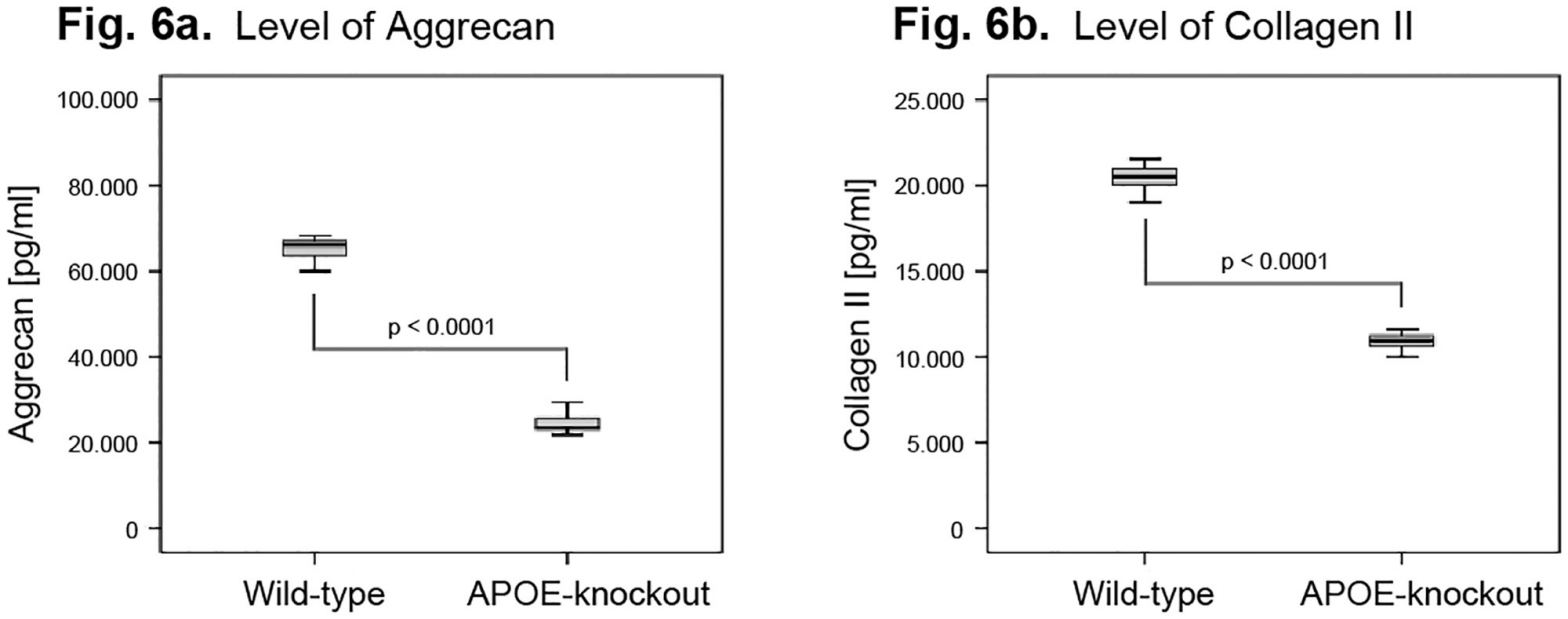
Declined aggrecan and collagen II levels in NP cells of APOE-knockout rabbits. NP cells of APOE-knockout and wild-type rabbits were isolated and 1 x 10^6^ cells were cultured in alginate gel for four weeks. The levels of the main matrix proteins were determined in 100 μg total protein extracts using ELISA and data were statistically analysed. Box plots with whiskers min. to max. show aggrecan levels (Fig 6a) and collagen II levels (Fig 6b).

The levels of the major IVD matrix proteins aggrecan, collagen II and collagen I were examined in NP cells of APOE-knockout and wild-type rabbits. An extremely decreased concentration level of aggrecan was detected in NP cells of APOE-knockout rabbits with mean concentration level of 24526 ± 2165 pg/ml, and the recorded mean concentration level in wild-type was 65087 ± 2552 pg/ml (P<0.0001) (Fig 6a). Moreover, the concentration level of collagen II in in NP cells of APOE-knockout rabbits was also diminished as compared to that in wild-types. Their mean levels amounted 10898 ± 450 pg/ml and 20526 ± 862 pg/ml respectively (P<0.0001) (Fig 6b). The concentration level of collagen I was not traceable in NP cells of both APOE-knockout and wild-type rabbits, though the kit detection limit was below 190 pg/ml.

## Discussions

Degenerative intervertebral disc disease is described as a biological disintegration process of IVD matrix that can be promoted by various factors that may cooperate with each other, such as genetical, nutritional and inflammatory factors. It has been previously shown that adult IVDs have significantly lower cell population density than IVDs of children. This is due to the fact that the concentration levels of glucose and oxygen decrease in the adult IVDs, as blood supply and diffusion decrease during aging. The declined nutrient supply into adult IVDs promotes harsh changes of the cell environment predominantly in the center of the IVDs [17, 40–42].

Degeneration of IVDs becomes apparent as early as the second decade of life. Comparable to IVD degeneration, atherosclerosis also starts to develop early in adult age. In almost the same manner the intensification of IVD degeneration and atherosclerosis in the abdominal aorta and aortic bifurcation, takes place between 44 and 64 years of age [13, 26, 43–44]. The paired lumbar and the middle sacral arteries that branch off from the abdominal aorta or aortic bifurcation are responsible for the nutrient supply to the lumbar vertebrae and IVDs [22–25]. Obstruction of nutrient supply into IVDs aided by atherosclerosis could change the metabolic and cellular environment particularly in the center of IVDs. NP cells that populate the center of IVDs play a fundamental role in maintenance of IVDs by orchestrating the expressions and activities of numerous inflammatory, catabolic, anti-catabolic and anabolic factors [15, 27, 35–36]. Degenerative changes in IVDs are associated with imbalances of these factors. Therefore, targeting their imbalances is an essential and a challenging task for establishing *in vivo* gene therapeutic approaches. For that reason, we examined the cell proliferation and the protein expression pattern that affect the NP tissue in APOE-knockout rabbits, which were recently characterized by structural deterioration of the IVD matrix that mimics the symptoms of advanced IVD degeneration in humans [34].

APOE-knockout rabbits showed decreased numbers of viable cells in directly isolated NP tissues as compared to that in wild-type rabbits (Fig 2a). The loss of cells in NP tissues of APOE-knockout rabbits by about 42% could be due to the obstructed nutrient transport mediated by atherosclerosis in the abdominal aorta and the lumbar arteries. The results support the data of the previous *in vitro* and *in vivo* studies that displayed the critical effect of nutrient deprivation on the maintenance of IVD cell viability and anabolic processes [34, 41]. The loss of cells in NP may indicate one of the fundamental causes for the lack of matrix regeneration. Nonetheless, NP cells isolated from the wild-type and APOE-knockout rabbits showed under similar *in vitro* culture conditions equivalent proliferation rates (Fig 2b). The results are consistent with our previous findings that showed comparable *in vitro* proliferation rates of NP cells, which were isolated from human IVDs of different degeneration grades [35–36]. The intact in vitro proliferation potential of NP cells that are isolated from degenerative IVDs could be beneficial for approaches of cell transplantation therapies, which are based on genetically modified autologous NP cells.

Moreover, enhanced levels of the inflammatory cytokines TNF-α, TNF-α R1, IL-1β and IL-1 R were confirmed in NP cells of APOE-knockout rabbits (Fig 3). These could evidently boost the expression of catabolic factors and promote degradation of the IVD matrix [45–46]. The data are in line with the results obtained in human NP cells, which showed higher levels of TNF-α, TNF-α R1, IL-1β, IL-1 R and IL-1 Ra in degenerative than non-degenerative IVDs; and the levels increased with the severity of degeneration. Moreover, treatments of human NP cells with recombinant TNF-α or IL-1β have been shown to enhance the expression levels of the catabolic factors ADAMTSs and MMPs [45–46].

Despite the fact that we found relatively high levels of ADAMTS-4 and ADAMTS-5 in NP cells of wild-type rabbits, we detected critically enhanced levels of ADAMTS-4 (3.3 fold) and ADAMTS-5 (2.3 fold) in NP cells of APOE-knockout rabbits (Fig 4a and 4b). The results apparently specify the importance of ADAMTS-4 and ADAMTS-5 as therapeutic targets, whereby ADAMTS-4 appears to be the predominant one. In previous studies overexpression of ADAMTS-4 and ADAMTS-5 has been shown to aggravate the aggrecan degradation and inhibition of ADAMTS-4 by shRNA could significantly increase the levels of aggrecan and collagen II [47–49]. From all tested MMPs, NP cells of APOE-knockout rabbits showed enhanced levels of MMP-1 and MMP-3, whereas MMP-3 exhibited by far the highest level (Fig 4c and 4d). However, the enhanced levels of MMP-1 and MMP-3 were counteracted by the even higher level of TIMP-1 (Fig 5a), which is known as the main inhibitor of MMPs [50]. In contrast, we determined in NP cells of APOE-knockout rabbits a considerably lower level of TIMP-3 (Fig 5c), the most important inhibitor of ADAMTs [50]. Our results indicate that inhibition of ADAMTs, rather than MMPs, would be the more effective approach to impede progressive degeneration of IVDs. The results also support the previous findings of the research in human IVDs, which recommend the imbalances between ADAMTSs and TIMPs as crucial targets to decelerate the enhanced degradation of matrix proteins in NP, such as aggrecan and collagen II [35–36, 49–50].

In addition to that, our data exhibited imbalances between the imperceptible levels of anabolic factors (Tab.1) and the critically enhanced levels of catabolic factors in NP cells of APOE-knockout rabbits, which highlight a decrease in stimulation of matrix synthesis and an increase in promotion of matrix degradation. In line with this, we detected significantly diminished levels of aggrecan and collagen II in NP cells of APOE-knockout rabbits (Fig 6).

In conclusion, APOE-knockout in rabbits could induce accelerated IVD degeneration that could be characterized by molecular and structural changes mimicking the symptoms of advanced IVD degeneration in humans. The results apparently point out that degenerative change in IVDs could be caused not only by loss NP cells but also by unfavourable phenotypic changes in NP cells. NP cells of APOE-knockout rabbits exhibited enhanced levels of inflammatory catabolic cytokines, imbalances between catabolic and anti-catabolic factors along with imbalances between catabolic and anabolic factors, that emulate the results found in human NP cells of degenerative IVDs.

Therefore, APOE-knockout rabbits could be used as a promising animal model for therapeutic approaches of degenerative disc disorders. They can be seen as an advantageous *in vivo* model, as they can be used in studies of both disc disorder and atherosclerosis. Moreover, they can bypass the standard surgical interventions that are commonly applied for induction of accelerated disc degeneration in other research animals, and so contribute to the 3Rs.

APOE-knockout rabbits can be used as a new model to set up a stable, less immunogenic and non-pathogenic ex vivo gene therapy system to enhance the regeneration potential of NP cells and advance the treatment strategies of degenerative disc disorders. For that degenerative NP cells can be isolated from NP tissue of APOE-knockout rabbits and transduced in culture by recombinant AAV6 carrying the relevant therapeutic genes identified here, as we recently established in degenerative human NP cells [51–52]. The genetic modified NP cells can be transplanted into NP of APOE-knockout rabbits and the course of IVD regeneration can be assessed using magnetic resonance imaging, immunohistological and biomolecular techniques.

## Supporting information

S1 Checklist ARRIVE Guidelines Checklist. (PDF)

S1 File Statistics in Fig 2B. (PDF)
