# Supplementary material for: APOE-knockout in rabbits causes loss of cells in nucleus pulposus and enhances the levels of inflammatory catabolic cytokines damaging the intervertebral disc matrix
Source: PLoS One. 2019 Nov 21;14(11):e0225527. doi: 10.1371/journal.pone.0225527 (PMC6871866; doi:10.1371/journal.pone.0225527)
Supplement: S1 File — (PDF) [file pone.0225527.s002.pdf]

```
T-TEST PAIRS=VAR00003 WITH VAR00004 (PAIRED)
  /CRITERIA=CI(.9500)
  /MISSING=ANALYSIS.
```

T-Test

Paired Samples Statistics

		Mean	N	Std. Deviation	Std. Error Mean
Pair 1	Wild-type	3513561,333	24	96851,59549	19769,74914
	APOE-knockout	3464578,625	24	130332,7915	26604,06967

Paired Samples Correlations

		N	Correlation	Sig.
Pair 1	Wild-type & APOE-knockout	24	,695	,000

Paired Samples Test

		Paired Differences			
					95% Confidence ...
		Mean	Std. Deviation	Std. Error Mean	Lower
Pair 1	Wild-type - APOE-knockout	48982,70833	93957,39473	19178,97289	9307,98011

Paired Samples Test

		Paired ...			
		95% Confidence Interval of the ...			
		Upper	t	df	Sig. (2-tailed)
Pair 1	Wild-type - APOE-knockout	88657,43656	2,554	23	,018

```
EXAMINE VARIABLES=VAR00003 VAR00004
  /COMPARE VARIABLE
  /PLOT=BOXPLOT
  /STATISTICS=NONE
  /NOTOTAL
  /MISSING=LISTWISE.
```

Explore

Case Processing Summary

	Cases					
	Valid		Missing		Total	
	N	Percent	N	Percent	N	Percent
Wild-type	24	100,0%	0	0,0%	24	100,0%
APOE-knockout	24	100,0%	0	0,0%	24	100,0%

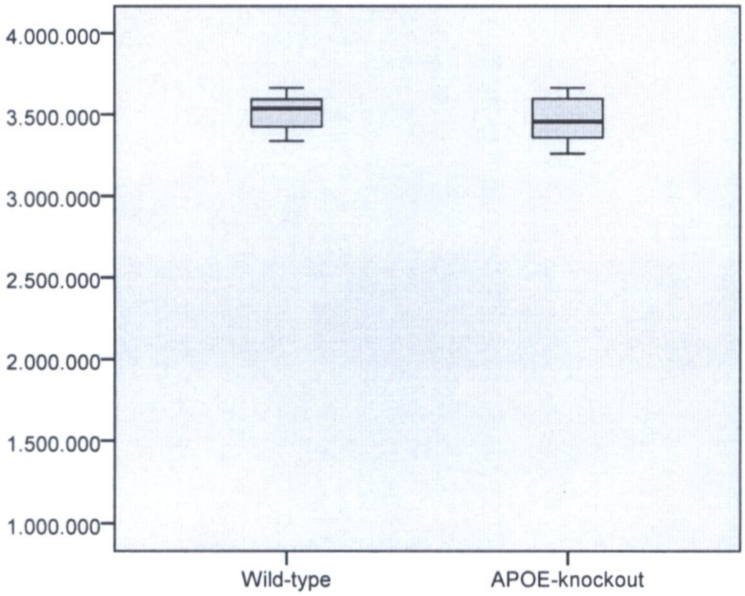


```
ONEWAY VAR00004 BY VAR00003
  /MISSING ANALYSIS.
```

Oneway

ANOVA

APOE-knockout

	Sum of Squares	df	Mean Square	F	Sig.
Between Groups	3,907E+11	23	1,699E+10	.	.
Within Groups	,000	0	.		
Total	3,907E+11	23			

```
ONEWAY VAR00003 BY VAR00004
  /MISSING ANALYSIS.
```

Oneway

ANOVA

Wild-type

	Sum of Squares	df	Mean Square	F	Sig.
Between Groups	2,157E+11	23	9380231548	.	.
Within Groups	,000	0	.		
Total	2,157E+11	23			
